# Balancing tradition and animal welfare: Adapting an animal-involving festival to climate change in Fukushima, Japan

**DOI:** 10.1016/j.onehlt.2025.101270

**Published:** 2025-11-12

**Authors:** Fumiue Harada, Morihito Takita, Kenji Shibuya

**Affiliations:** a Department of Internal Medicine, Soma Central Hospital, Soma, Fukushima, Japan; b Department of Disaster and Comprehensive Medicine, Fukushima Medical University, Fukushima, Japan; c Department of Radiation Health Management, Fukushima Medical University, Fukushima, Japan; d Medical Governance Research Institute, Minato-ku, Tokyo, Japan; e Medical Excellence JAPAN, Chuo-ku, Tokyo, Japan

**Keywords:** Climate change, Heat stress, Animal welfare, Cultural adaptation, One health, Japan, Festival safety

## Abstract

Climate change poses critical risks to humans and animals, particularly during outdoor events. The Soma Nomaoi Festival in Fukushima, Japan, with a thousand-year history involving horses and riders, has become vulnerable to rising summer temperatures. In 2023, during a record high of 35.2 °C, 83 heatstroke cases occurred, including 74 among spectators and nine among horse riders, with two horse fatalities and 111 horses requiring medical care. In response, organizers rescheduled the 2024 festival to May, resulting in a maximum temperature of 24 °C. Human heatstroke cases dropped by 83 % (to 18 cases), and equine cases fell from 111 to 38. Although injuries from heightened horse activity increased, overall welfare improved. This case illustrates how traditional festivals can adapt to climate change while preserving cultural integrity. The experience demonstrates the value of the One Health approach in balancing cultural tradition, human safety, and animal welfare.

## Graphical abstract

**Figure:**
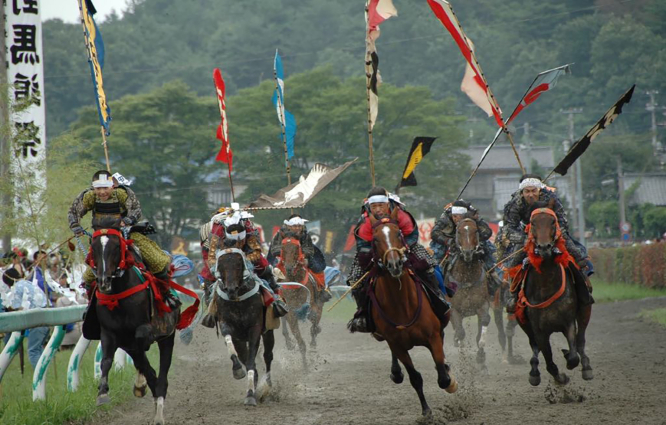

## 1 Introduction

Climate change is a critical global health challenge, producing more frequent extreme weather events such as droughts, floods, and heatwaves [1]. These conditions affect not only human health but also animal health and welfare, creating complex risks that demand integrated responses under the One Health framework. Mammals, in particular, are vulnerable to heat stress due to their high metabolic rates [2]. Rising summer temperatures are forcing a reassessment of traditional and religious events that involve close interaction between humans and animals.

Globally, the “San Fermín Festival” in Spain, known for the Running of the Bulls, has been criticized for safety risks to both humans and animals, with social media amplifying public scrutiny. Likewise, the Hajj pilgrimage in Saudi Arabia has repeatedly confronted challenges of mass gatherings during extreme heat; in June 2024, more than 1300 pilgrims died of heat-related illness despite preventive measures [3]. These examples illustrate the urgent need to adapt long-standing events to protect participants.

The Soma Nomaoi Festival is primarily a cultural event rooted in Shinto rituals. While it contains religious elements, it functions today mainly as a community-based cultural celebration rather than a purely religious observance. This distinction has made rescheduling feasible without conflicting with fixed religious calendars.

In Japan, the Soma Nomaoi Festival in Fukushima Prefecture represents a unique cultural heritage where horse-riding warriors reenact samurai cavalry practices. With origins over a thousand years ago, the festival integrates Shinto rituals, community identity, and equestrian traditions, attracting more than 120,000 visitors annually. However, rising summer temperatures in Tohoku—exacerbated by the foehn phenomenon, a hot and dry downslope wind—have raised serious safety concerns. This Short Communication reports how the festival adapted its schedule to climate change and evaluates outcomes for both humans and horses.

### 1.1 Case description: Soma Nomaoi Festival and climate risk

Traditionally held in late July, the Soma Nomaoi Festival has endured disruptions in the past, including scale-downs after the 2011 Great East Japan Earthquake and modifications during the COVID-19 pandemic. Yet climate change has presented a new and persistent challenge.

In 2023, Fukushima experienced a record high of 35.2 °C during the festival. Emergency medical teams recorded 83 cases of heat-related illness: 74 spectators and nine riders. Two horses died—one confirmed from heatstroke, another from unknown causes—and 111 horses required veterinary care, primarily for hyperthermia. These events intensified public debate about animal welfare and the sustainability of holding the festival at midsummer.

The review committee, balancing tradition and welfare, decided to reschedule the festival to a cooler period in 2024, from May 24–27. This was the first change in the festival's thousand-year history directly attributed to climate change.

### 1.2 Outcomes of rescheduling

In 2024, the maximum temperature at the main venue (37°37′27.15“ N, 140°57’31.28” E) was 24 °C, a full 11 °C lower than the previous year. The impact was striking:

Human heatstroke cases decreased by 83 %, with only 18 requiring medical assistance.

Equine medical interventions decreased from 111 to 38, of which 34 were heat-related.

However, medical consultations for trauma (bruises and injuries) rose to 21, likely linked to heightened horse activity during breeding season.

These data highlight the trade-offs inherent in rescheduling but underscore a net gain in safety and welfare for both humans and animals.

## 2 Discussion

The Soma Nomaoi case offers three important insights:

Cultural adaptation as climate response. Adjusting centuries-old traditions is difficult, but the decision to prioritize human and animal welfare demonstrates cultural resilience. By rescheduling, organizers preserved the festival's integrity while reducing health risks. This aligns with the One Health principle of integrated, multidisciplinary solutions.

Animal welfare as a central concern. The death of horses in 2023 galvanized public discourse on ethical responsibilities. In the modern era, it is increasingly unacceptable for animals to suffer or die for cultural practices. By moving the event, organizers recognized animal welfare as inseparable from human cultural values. Public health co-benefits. Protecting horses simultaneously reduced human medical emergencies. This reinforces the interdependence of human, animal, and environmental health, and provides a model for other regions facing climate-related festival risks. The 2024 adaptation also coincided with the end of an El Niño event, known to amplify heatwaves in Japan. Historically, subsidence of El Niño has led to extreme summers, as in 2010 and 2018. This context suggests that rescheduling will become increasingly necessary as climate variability intensifies. Other large-scale events illustrate the difficulty of adaptation. The Olympics must balance athlete safety with broadcast and commercial interests, while religious gatherings such as the Hajj are fixed to lunar calendars, limiting flexibility [4,5]. Local festivals like Soma Nomaoi may be more adaptable but also face challenges because they are interwoven with agricultural and community life. Nevertheless, the Fukushima example shows that even thousand-year traditions can evolve in response to climate change.

## 3 Conclusion

The rescheduling of the Soma Nomaoi Festival in 2024 represents a landmark adaptation to climate change, reducing human heatstroke cases by 83 % and improving equine welfare. While some injury risks increased, the overall balance favored safety and sustainability. This case demonstrates the potential of traditional events to embrace the One Health framework, protecting both humans and animals in an era of rising climate threats. Future research should monitor long-term impacts of rescheduling, including community acceptance, tourism economics, and equine welfare indicators. As global warming progresses, proactive adaptation of cultural events will be essential to safeguard lives while preserving heritage.

## Author statement

All authors listed on this manuscript have made substantial contributions to the conception and design of the work, and/or the acquisition, analysis or interpretation of the data; have drafted or revised the manuscript critically for important intellectual content; and have given final approval of the version to be published. All authors agree to be accountable for all aspects of the work in ensuring that questions related to the accuracy or integrity of any part of the work are appropriately investigated and resolved.

Each author declares that the work described has not been published previously (except in the form of an abstract or as part of a published lecture, review, or thesis), is not under consideration for publication elsewhere, and its publication is approved by all authors and tacitly or explicitly by the responsible authorities where the work was carried out.

If the study involved humans and/or animals, the Authors declare that appropriate ethical approval (by a recognized ethics committee for human subjects or an institutional animal care and use committee) was obtained and is clearly stated in the Methods section. Relevant details (e.g., approval number or protocol) are provided.

Authors also affirm that they have disclosed all sources of funding and any potential conflicts of interest. A separate “Competing interests” section is included in the manuscript.

## CRediT authorship contribution statement

**Fumiue Harada:** Writing – review & editing, Writing – original draft. **Morihito Takita:** Writing – review & editing. **Kenji Shibuya:** Writing – review & editing.

## Declaration of competing interest

The authors declare that they have no known competing financial interests or personal relationships that could have appeared to influence the work reported in this paper. Declaration of generative AI and AI-assisted technologies in the writing process: During the preparation of this work, the authors used ChatGPT (OpenAI) to improve the readability and language of the manuscript. After using this tool, the authors reviewed and edited the content as needed and take full responsibility for the content of the published article.

## Data Availability

Data will be made available on request.
